# Using three-dimensional visualization as an optimal tool to plan and validate an aortopexy in a congenital heart disease patient with severe tracheal stenosis

**DOI:** 10.1093/icvts/ivab315

**Published:** 2021-11-05

**Authors:** Torben Kehl, Victoria van Rüth, Julius Matthias Weinrich, Michael Hübler

**Affiliations:** 1 Clinic for Children’s Heart Medicine, University Heart & Vascular Center, University Hospital Hamburg-Eppendorf, Hamburg, Germany; 2 Section of Pediatric Radiology, Department of Diagnostic and Interventional Radiology and Nuclear Medicine, University Hospital Hamburg-Eppendorf, Hamburg, Germany

**Keywords:** 3D visualization, Tracheal stenosis, Congenital heart disease, Aortopexy

## Abstract

We present a patient with severe tracheal stenosis resulting from a compression by the innominate artery 6 months after an arterial switch operation in a dextro-transposition of the great arteries. Segmentation and three-dimensional (3D) visualization were derived from a contrast-enhanced dual-source computed tomography and post-processing was performed using a dedicated open-source platform (3D Slicer). Post-processing allowed a comprehensible visualization of the relationship of the innominate artery to the trachea when compared to standard computer tomography reformations. Finally, the surgical approach to move the innominate artery anteriorly in order to relieve the tracheal obstruction was emphasized based on the improved 3D visualization of the actual pathology. An effective aortopexy could be performed and the postoperative result was confirmed by a second 3D visualization. About 3 months of follow-up, the patient is completely asymptomatic. Three-dimensional visualization offers excellent opportunities for diagnosis, treatment planning and follow-up in patients with a vascular-related tracheal stenosis in the context of congenital heart disease.

## INTRODUCTION

Tracheal stenosis in paediatric patients may be related to a variety of aetiologies [1]. In case of vessel-related tracheal compression aortopexy provides an established treatment approach [2].

## CASE REPORT

We present a 6-month-old patient with severe distal tracheal stenosis related to compression by a left-sided innominate artery (right aortic arch) after neonatal arterial switch operation. The patient developed progressive expiratory stridor and cyanotic spells. Biplane chest X-ray and echocardiography were unremarkable. Diagnosis of tracheal compression by the innominate artery was confirmed using a third-generation dual-source computer tomography scanner (Somatom Force^®^, Siemens Healthineers AG, Erlangen, Germany) with high temporal (111 ms) and spatial resolution. The dedicated cardiac protocol [3] has been reported in detail previously and resulted in a very low radiation exposure (effective dose 0.36 milli-Sievert).

Three-dimensional (3D) image reconstruction was carried out by an open-source platform for analysis and display of medical imaging data (3D Slicer) [4]. Hounsfield unit-based thresholds and manual processing tools allowed for a precise 3D reconstruction of the maximal tracheal compression as well as the surrounding anatomical structures and the distance between the innominate artery and the sternum.

## RESULTS

**Figure 1:**
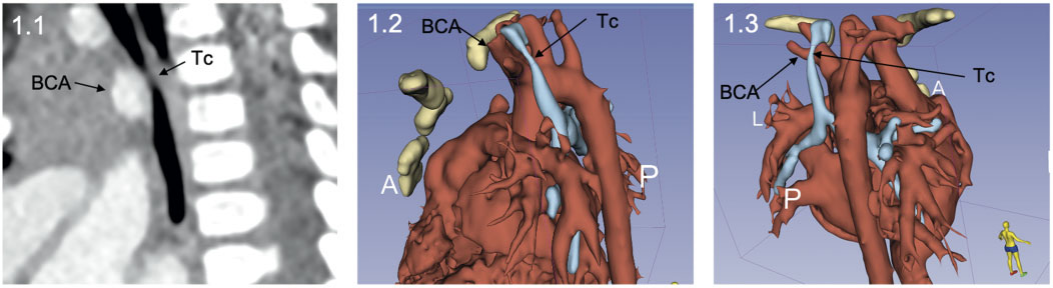
Preoperative computer tomography scan [sagittal slice (1.1) through the maximum of the tracheal stenosis] and three-dimensional visualization (1.2–1.3) of the sternum, the whole heart, the aortic arch with the left-sided innominate artery (BCA) and the compressed Tc shown from different perspectives. BCA: brachiocephalic artery; Tc: trachea.

**Figure 2:**
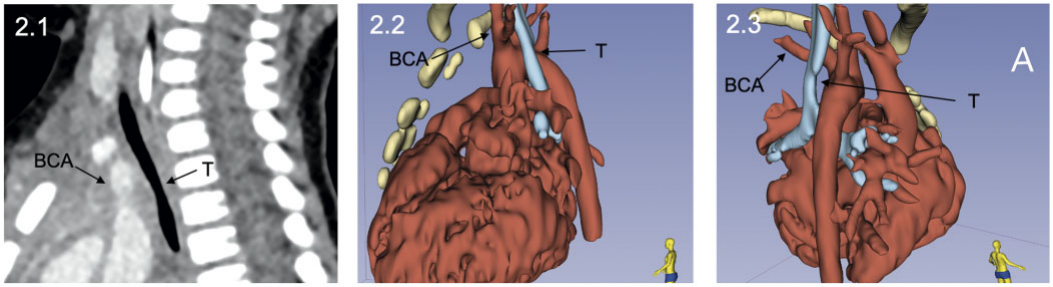
Postoperative computer tomography scan [sagittal slice (2.1) with enlarged diameter of the trachea and three-dimensional visualization (2.2–2.3) of the sternum, the whole heart, the aortic arch with the left-sided innominate artery (BCA) and the decompressed T] shown from different perspectives. BCA: brachiocephalic artery; T: trachea.

The applied 3D reconstruction provided excellent visualization of the pathology as well as the surrounding anatomy emphasizing the surgical approach to move the innominate artery anteriorly in order to relieve the tracheal obstruction (Fig. 1 and Video 1). The 3D reconstruction allowed us to target planning for dissection and suspension of the left innominate artery on the left side of the sternum. The success of the procedure after sternal closure was proven by parallel tracheoscopy. During early postoperative course, the patient had some minor residual stridor. A follow-up computer tomography scan (effective dose 0.33 milli-Sievert) and a repeated 3D reconstruction (Fig. 2 and Video 2) ruled out tracheal compression, thus, an additional tracheoscopy was waived. After 5 months of follow-up, the patient is now completely asymptomatic.

## DISCUSSION

Tracheal stenosis in paediatric patients may be related to a variety of aetiologies [2]. Apart from oesophageal atresia, an atypical course of the innominate artery is one of the more common causes [2]. In the presented patient, tracheal stenosis resulted from compression by the left-sided innominate artery of a right aortic arch 6 months after arterial switch operation.

Typically, in complex anatomy cross-sectional imaging is the reference of standard [2]. Post-processing software for segmentation and 3D visualization allows for a much more precise visualization, particularly from the surgeon’s perspective.

Unexpected postoperative findings in this setting, such as mild residual stridor, were verified by a second 3D reconstruction and clear visualization of the postoperative situs. While a tracheoscopy alone would have only allowed to evaluate the tracheal stenosis, the follow-up computer tomography not only visualized the decompression of the trachea but also the anteriorly extended aortic arch and the surrounding structures as well.

However, 3D reconstruction may result in a loss of information: The connecting tissue between the aortic arch and the trachea was not segmented and, thus, is not displayed. As a result, the distance between these anatomic structures appears wider in 3D than in reality.

In addition to the 3D visualization shown here, 3D printing is an alternative for preoperative planning. Improvements in the preoperative planning and postoperative outcomes could be shown [5].

However, the here presented approach has the advantage of faster availability and lower costs due to the open-source platform.

## CONCLUSION

Three-dimensional visualization offers excellent opportunities for diagnosis, treatment planning and follow-up in patients with a vascular-related tracheal stenosis in the context of congenital heart disease.
